# Novel variants in the *ACTA2* and *MYH11* genes in a Cypriot family with thoracic aortic aneurysms: a case report

**DOI:** 10.1186/s12881-018-0728-0

**Published:** 2018-12-07

**Authors:** Anna Keravnou, Evy Bashiardes, Kyriaki Michailidou, Marinos Soteriou, Areti Moushi, Marios Cariolou

**Affiliations:** 1 Department of Cardiovascular Genetics and The Laboratory of Forensic Genetics, The Cyprus Institute of Neurology and Genetics, Nicosia, Cyprus; 2 Cyprus School of Molecular Medicine, The Cyprus Institute of Neurology and Genetics, Nicosia, Cyprus; 3 Department of Electron Microscopy/Molecular Pathology, The Cyprus Institute of Neurology and Genetics, Nicosia, Cyprus; 4 American Medical Center, Nicosia, Cyprus

**Keywords:** Aortic aneurysm, *ACTA2*, *MYH11*, Cyprus, Targeted next generation sequencing

## Abstract

**Background:**

Thoracic aortic aneurysm (TAA) and/or thoracic aortic aneurysm and dissection (TAAD) is characterized by a considerable risk of morbidity and mortality of affected individuals. It is inherited in an autosomal dominant pattern and the 20% of patients with non-syndromic TAA have a positive family history. To date, the genetic basis of Cypriot patients with TAA has not been investigated. The purpose of this case report is to determine underlying genetic cause in this Cypriot family with TAA.

**Case presentation:**

In this report we present a patient with hyper-acute onset chest and back pain diagnosed with Type A Aortic Dissection with severe aortic valve regurgitation, who underwent emergency aortic surgery and Bentall procedure. Further investigation of the patient’s family was undertaken where both parents and an additional child were also found to be affected. A targeted sequencing panel including genes with known association to TAA was used to identify causative mutations in the index patient. Massively Parallel Sequencing results identified a frameshift deletion c.363_367del GAGTC, p.Met121Ilefs*5 in the *ACTA2* gene and a non-synonymous variant c.3234C > G, p.Ile1078Met in the *MYH11* gene. The presence or absence of these variants in the index patient and other family members was verified by Sanger sequencing. To our knowledge, this is the first report of a Cypriot family case diagnosed with TAA presented by two novel variants one in the *ACTA2* and the other in the *MYH11* genes.

**Conclusions:**

We describe two novel variants in a Cypriot family with TAA that are potentially pathogenic, highlighting the importance of molecular genetic evaluation in families with TAA. These results may prove useful for screening purposes in Cypriot patients with non-syndromic familial TAA facilitating early identification of atrisk family members and direct intervention.

**Electronic supplementary material:**

The online version of this article (10.1186/s12881-018-0728-0) contains supplementary material, which is available to authorized users.

## Background

Thoracic aortic aneurysms (TAAs) and dissection (TAAD) is an aortopathy that can cause aortic rupture which is associated with a considerable risk of morbidity and mortality. TAA is a complex, heterogeneous and often lethal disease that predominantly displays an autosomal dominant pattern of inheritance [1, 2]. The majority of the patients (95%) do not have any symptoms until a major event occurs, such as aortic dissection or rupture [3–5]. It may be classified as syndromic or non-syndromic, including familial TAA. It is estimated that approximately 20% of patients with non-syndromic TAA have a positive family history [3]. Familial studies showed that there is a 10-fold increased incident rate in first-degree relatives with a family history of TAA. Therefore, screening of first-degree relatives should be introduced as part of clinical practice [6].

With recent advances in Massively Parallel Sequencing (MPS), new variants associated with TAA clinical phenotype have been identified that relate to the clinical phenotype [7–9]. To date, 30 causative genes (Additional file 1) [10] that contribute to the development of TAA have been identified with the majority being related to genes encoding proteins involved in the smooth muscle cell contractile apparatus (e.g. *ACTA2*, *MYH11*, *MYLK*, and *PRKG1*), extracellular matrix (e.g. *FBN1*), or the transforming growth factor-β signaling pathway (e.g. *TGFB2*, *TGFBR1*, *TGFBR2*, and *SMAD3*). Therefore, the identification of novel variants in genes associated with TAA is considered an area of progress that may lead to earlier detection and treatment of the disease [11, 12].

In the current study our aim was to identify any possible genetic cause, for the first time, in a Cypriot familial case of TAA in which two parents and the two children were affected. When preparing this case report the CARE Guidelines: Consensus-based Clinical Case Reporting Guideline Development were taken into account [13].

## Case presentation

The study was approved by the Cyprus National Bioethics Committee and informed, written consent was obtained from all participants.

The patient under discussion is a 30 year old male, weight lifter with hyper-acute onset chest and back pain. Upon initial evaluation, the patient did not have any manifestation of any phenotypic findings (i.e. iris flocculi, livedo reticularis, aortic valve abnormalities) hinting towards connective tissue disorders or aortic pathology. Based on echocardiography and chest CT findings the patient was diagnosed with a Type A Aortic Dissection with severe aortic valve regurgitation. The patient was then emergently intubated and was transferred to the American Medical Center (AMC) in Nicosia, Cyprus where he had emergency aortic surgery and Bentall procedure. No angiography was performed due to the fact that the patient had aortic dissection and was an emergency. Intraoperative findings showed a 70 mm diameter aortic root aneurysm with dissection extending from the sinotubular junction to the aortic root. The aortic annulus was extensively dilated and the aortic dissection extended down the origin of the left main coronary artery involving also the aortic valve commissures that resulted in severe aortic valve regurgitation. There was no evidence of involvement of the distal ascending aorta and the aortic arch.

**Fig. 1:**
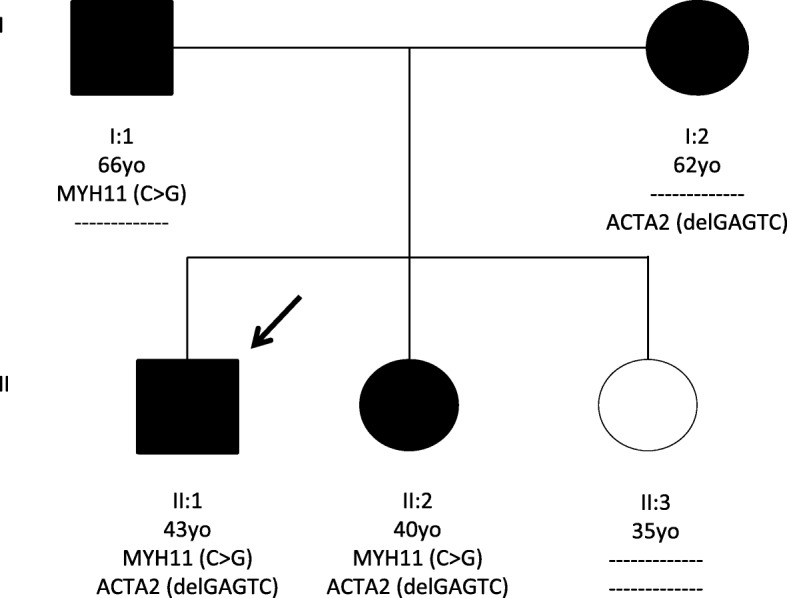
Pedigree of the family with c.3234C > G, p.Ile1078Met variant in *MYH11* gene and c.363_367del GAGTC, p.Met121Ilefs*5 variant in *ACTA2* gene. The individual II:2 on initial screening had normal aortic measurements and after genetic screening was re-evaluated and found to have a mild dilation. Proband is indicated with an arrow; Black-filled symbols represent affected individuals with TAA; Age of the family members and their genotypes are listed below the symbols; Dotted lines indicate the absence of the mutation

Further evaluation of the patient’s family showed that several family members had ascending aortic aneurysms. The patient’s father, a 60-year-old male had an asymptomatic aortic root and ascending aortic aneurysm of 52 mm that also required aortic valve, aortic root and ascending aortic replacement (Bentall procedure). Pre-operatively thefather had CT chest coronary angiography that confirmed the presence and dimensions of the aneurysm and coronary angiography did not show any coronary artery disease. The patient’s mother, a 56-year-old female was also screened with echocardiography that showed ascending aortic dilatation, 42 mm in diameter. Subsequent screening of the siblings, two sisters at the ages of 22 and 34 respectively did not show any abnormalities. Following the genetic screening of the index patient, his parents and his siblings, a follow-up was recommended for both his sisters. Six years after the initial screening of the sisters, the 34-year-old was found to have a mild dilation of the ascending aorta (39 mm) by echocardiography with a 4–5 mm increase in diameter in comparison with the previous echocardiography results. The pedigree of the family is demonstrated in Fig. 1. The index patient’s and the family’s medical diagnosis and interventions are shown in Table 1.

**Table 1:** The index patient’s and the family’s medical diagnosis and interventions

	Relevant Past Medical History and Interventions				
	No past medical history reported by the index patient whose symptoms and diagnosis were initiated in 2005				
Dates	Study Project	Sequence Variants	Summaries from Initial and Follow-up Visits	Diagnostic Testing	Interventions
2005	Index patient	MYH11:c.3234C > G, p.Ile1078Met ACTA2:c.363_367delGAGTC, p.Met121Ilefs*5	Hyper-acute onset chest and back pain	Echocardiography and CT Chest	Bentall procedure
2016			Follow-up visit	Echocardiogram	No further intervention as no significant change found
2017			Counseling visit	Genetic testing	Novel variants detected in *ACTA2* and *MYH11* genes
2012	Father	MYH11:c.3234C > G, p.Ile1078Met	Asymptomatic	Echocardiography and CT Chest coronary angiography	Bentall procedure
2015			Follow-up visit	Henoch-Schonlein purpura in low extremities, Kidney failure	Hemodialysis
2017			Follow-up visit	Echocardiogram	No further intervention recommended
2017			Counseling visit	Genetic testing	Novel variant detected in *MYH11* gene
2012	Mother	ACTA2:c.363_367delGAGTC, p.Met121Ilefs*5	Asymptomatic	Echocardiogram	Ascending aortic dilatation
2015			Follow-up visit	Echocardiogram	No significant change found
2017			Counseling visit	Genetic testing	Novel variant detected in *ACTA2* gene
2005	Sister #1	MYH11:c.3234C > G, p.Ile1078Met ACTA2:c.363_367delGAGTC, p.Met121Ilefs*5	Asymptomatic	Echocardiogram	Normal aortic size
2012			Follow-up visit	Echocardiogram	Normal aortic size
2017			Counseling visit	Genetic testing	Novel variants detected in *ACTA2* and *MYH11* genes
2017			Follow-up visit	Echocardiography	Mild dilation of the ascending aorta
2005	Sister #2		Asymptomatic	Echocardiogram	Normal aortic size
2012			Follow-up visit	Echocardiogram	Normal aortic size
2017			Counseling visit	Genetic testing	No novel variants detected in *ACTA2* and *MYH11* genes

To understand any genetic implication in the cause of TAA of this family, targeted sequencing was performed on DNA samples from the father and the son. Whole blood samples were used to extract DNA using the QIAamp Blood Midi Kit (Qiagen, Hilden, Germany) according to the manufacturer’s instructions.

**Fig. 2:**
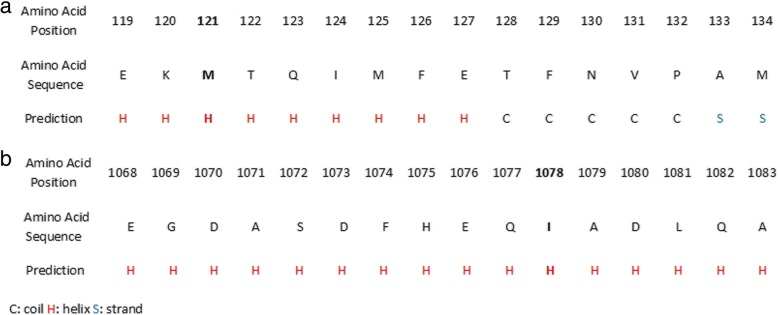
The predicted secondary structure of **a**. ACTA2 and **b**. MYH11 proteins indicating, the location of the amino acids Met121 (bold) and Ile1078 (bold) respectively, on a helix structure where the variants (p.Met121Ilefs*5) and (p.Ile1078Met) occur using I-TASSER modeling

The TruSight Cardio Sequencing panel (Illumina, San Diego, CA, USA) which includes 16 genes, the most predominant, with known association to familial aortic aneurysm was initially used and paired-end sequencing was performed on an Illumina Miseq platform at our Institute. Our results showed that the two samples (son and father) had an average depth of coverage of 117x and 108x respectively for the *MYH11* region and an average depth of coverage of 135x and 125x for the *ACTA2*. Raw variants were filtered to include only those variants with a coverage depth greater than 20× and a Quality score greater than 30. The Illumina run generated 94.8% of base calls having >Q30. We followed the Genome Analysis Toolkit (GATK) best practices for variant calling. Sequencing reads were aligned to the human reference genome build GRCh37/hg19 using Burrows Wheeler Aligner (BWA) software. Different GATK programs were used for local realignment and base recalibration and the SAMtools (Sequence Alignment/Map) pipeline was used to retrieve per-base read depth information. Calling was performed using Haplotype Caller from the GATK. The called Small Nucleotide Variants (SNVs) and Insertions and Deletions (InDels) were annotated using the Annotate Variation (ANNOVAR) [14] software. The variants were then filtered using the GATK best practices (https://software.broadinstitute.org/gatk/), focusing on rare (minor allele frequency < 0.05) and known pathogenic variants. MPS results identified a frameshift deletion c.363_367del GAGTC, p.Met121Ilefs*5 in the *ACTA2* gene (Genbank transcript ID: NM 001613) and a non-synonymous variant c.3234C > G, p.Ile1078Met in the *MYH11* gene (Genbank transcript ID: NM 001040114). In order to predict the pathogenicity of the novel variants, the in silico software of the computational algorithms SIFT (Sorting Intolerant from Tolerant) [15] (http://sift.jcvi.org), PolyPhen2 (Polymorphism Phenotyping) [16] (http://genetics.bwh.harvard.edu/pph2), Mutation Taster [17] (http://www.mutationtaster.org), PROVEAN (Protein Variation Effect Analyzer) [18] (http://provean.jcvi.org) and VEP (Variant Effect Predictor) [19] (https://asia.ensembl.org/Tools/VEP) were used. All five different prediction tools (SIFT: Deleterious PolyPhen2: Probably Damaging, Mutation Taster: Disease causing, PROVEAN: Deleterious, VEP: High) presented a consistent result describing the p.Met121Ilefs*5 (c.363_367delGAGTC) variant as having a detrimental/deleterious effect. These predictions, suggest that this variant has a significant effect on the function of *ACTA2* and the development of TAA. Regarding the p.IIe1078Met (c.3234C > G) variant in the *MYH11* gene, PolyPhen2 and PROVEAN prediction tools presented it as being a benign and neutral variant respectively while, VEP, SIFT and Mutation Taster suggested it as being a moderate, deleterious and disease causing variant respectively In addition, Iterative Threading ASSEmbly Refinement (I-TASSER) modeling [20–22], a protein structure prediction tool, was used to predict the secondary structure of the ACTA2 and MYH11 proteins. The results showed the presence of the amino acids Met121 and Ile1078 respectively, on a helix structure motif (Fig. 2). It is difficult to describe the protein function of the regions where these variants occur as the protein structure of ACTA2 and MYH11 is not completely known as yet after performing a search in UniProtKB and Protein Data Bank (PDB).

It is noteworthy that no known pathological sequence variants were identified in the affected individuals we studied. The TruSight Cardio sequencing panel led to the identification of a 5 bp frameshift deletion in the *ACTA2* gene (c.363_367del, p.Met121Ilefs*5) which results in a premature stop codon as well as a non-synonymous variant in the *MYH11* gene (c.3234C > G:p.I1078M) predicted as deleterious and disease causing by the in silico software of the computational algorithms used. Both variants were absent from public variant databases, such as the dbSNP (http://www.ncbi.nlm.nih.gov/SNP/), 1000 genome dataset (http://browser.1000genomes.org/index.html), Genome Aggregation Database (http://gnomad.broadinstitute.org), and Human Genetic Variation Browser (http://www.hgvd.genome.med.kyoto-u.ac.jp/) databases. With respect to the *MYH11* (c.3234C > G:p.I1078M) it is noteworthy that (c.3234C > T:p.I1078I) has been detected four times in the Genome Aggregation Database (GnomAD) and it is however synonymous (GnomAD, *n* = 282,728). Both the affected father and son displayed the novel heterozygous p.Ile1078Met variant in the *MYH11* gene while the son was also identified with the novel heterozygous p.Met121Ilefs*5 (c.363_367delGAGTC) variant in the *ACTA2* gene.

The identified variants were verified in the proband and other consenting family members (father, mother and two sisters) were screened by Sanger sequencing. PCR amplification was performed using 5 Units AmpliTaq DNA polymerase (Applied Biosystems, Foster City, CS). The *ACTA2* gene was initially amplified by PCR at 55 °C annealing temperature using the *ACTA2*-Forward: 5’ CTTTCAAGCTGTTCCTGTC 3′ and the *ACTA2*-Reverse: 5’ TGTGTTTCTCCTCTGTCC 3′ primers. The *MYH11* gene was amplified by PCR at 55 °C annealing temperature using the *MYH11*-Forward: 5’ AGTGAACAGGGTCGAGAAG 3′ and the *MYH11*-Reverse: 5’ TTTGCAGTGCGGCTAAAG 3′ primers. Amplified products were cleaned using QIAquick PCR Purification Kit (Qiagen, Hilden, Germany) and subjected to cycle sequencing according to the manufacturer’s instructions [BigDye Terminator v1.1 Cycle Sequencing Kit (Applied Biosystems]. The reactions were run on a 3130xL Genetic Analyzer (Applied Biosystems, Foster City, CA, USA) with the results analyzed via the Sequencing Analysis 5.2 Software (Applied Biosystems).

**Fig. 3:**
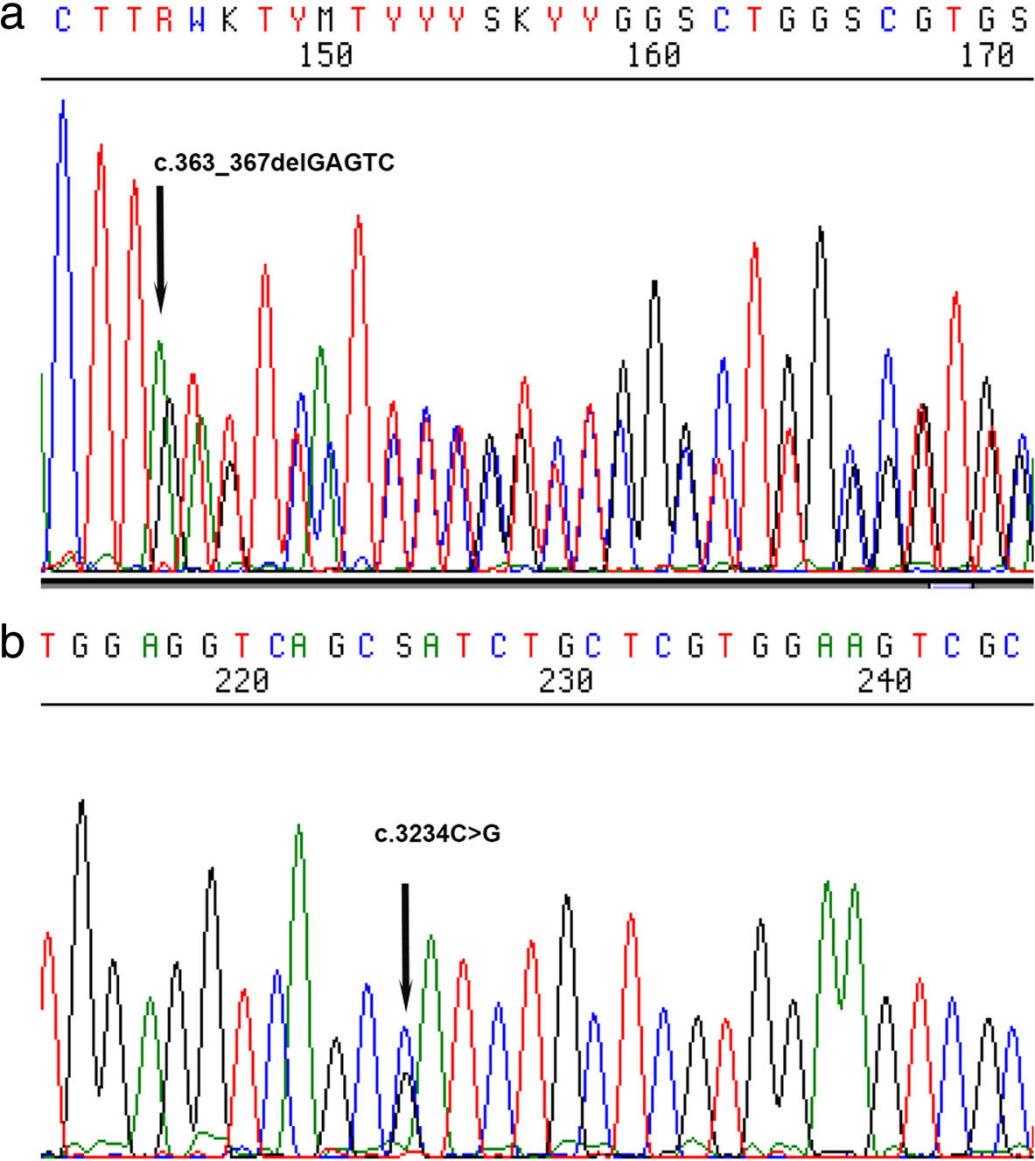
Representative Sanger sequencing of PCR products showing the index patient with the biallelic variants in **a**. *ACTA2* gene (c.363_367delGAGTC, p.Met121Ilefs*5) and **b**. *MYH11* gene (c.3234C > G, p.IIe1078Met)

Sanger sequencing analysis of the patients’ *ACTA2* and *MYH11* genes revealed that the p.IIe1078Met variant was inherited from the father and the p.Met121Ilefs*5 variant from the mother (Fig. 3). Results showed that the mother, the son and the 34-yo daughter had the p.Met121Ilefs*5 variant whereas the father, the son and the same daughter had the p.Ille1078Met variant. Overall, results showed that the affected son and the one daughter are carriers of both variants inherited one from each parent. The second daughter did not show any variants in either of the two genes.

In order to evaluate evidence for a sequence variant we followed the guidelines of the American College of Medical Genetics and Genomics (ACMG) [23]. The two variants (chr10:90703556_90703560delGAGTC and chr16:15831386 G > C) of *ACTA2* and *MYH11* genes are a deletion which results in a truncated transcript in exon 4 (c.363_367del GAGTC, p.Met121Ilefs*5) and a single base substitution in exon 26 (c.3234C > G, p.Ile1078Met), respectively. Combining the PVS1 criterion (predicted as a null variant) with 1 Moderate (PM2: Absence in population databases) classifies the ACTA2 variant as “likely pathogenic”. The MYH11 variant was interpreted as “variant of uncertain significance (VUS)” based on the following criteria: Absent in population databases (PM2), and computational evidence by different in silico tools applied supported a deleterious effect on the gene (PP3). For the p.Ile1078Met of MYH11, the small number of affected individuals (meiosis) was not enough to use the PP1 criteria. Although this variant is not found in GnomAD controls and is located on coiled-coil myosin heavy chain tail region, the evidence at this point is not enough to indicate that p.Ile1078Met is pathogenic according to ACMG standards. Therefore, we concluded that the variant in *ACTA2* is more likely to contribute to the TAA phenotype.

## Discussion and conclusions

TAAD is a potentially lethal disease and it is important to identify individuals who are at risk that can benefit from extensive surveillance and potentially undergo prophylactic treatment. Over the last decades with the implementation of MPS the understanding of gene mutations associated to TAA has greatly improved. As a result, it is expected that a higher number of genes and definitive pathogenic variants will be identified in the following years which will enable further investigation of the pathways that contribute to the onset of disease [24].

To the best of our knowledge this is the first report of a Cypriot family with non-syndromic familial TAA presented with two novel variants in *MYH11* and *ACTA2* genes. Previous studies reported that pathogenic sequence variants in *MYH11* (myosin heavy chain 11) and *ACTA2* (smooth muscle cytoskeletal protein actin alpha(α)-2) genes provide direct changes in contractile apparatus of aortic smooth muscle cells (SMC) which cause non-syndromic familial TAAD [1, 25]. Heterozygous mutations in *ACTA2* interfere towards the ability of arteries to stretch resulting in familial TAAD [1, 25–28]. Missense mutations in *ACTA2* gene account for the majority of familial TAADs and are responsible for 14% of non-syndromic TAAD [26, 29]. Mutations in *MYH11* gene result in the decrease of the elasticity of the aortic wall which is associated with familial TAAD [30]. Mutations in *MYH11* gene have also been associated with patent ductus arteriosus, one of the most common congenital cardiovascular malformations [30–32]. Pathogenic variants in *MYH11* gene are responsible for approximately 1% of heritable thoracic aortic disease [29].

In the current study, two novel variants were identified. Further in silico analysis, predicted that the novel p.Met121Ilefs*5 (c.363_367delGAGTC) and p.I1078M (c.C3231G:p.I1078M) variants in *ACTA2* and *MYH11* genes respectively, interfere with protein function and were characterized as possibly disease-causing variants. It was further predicted that the variant c.363_367del would have a more deleterious effect and hence be considered causative due to the introduction of a premature termination codon that would lead to nonsense-mediated mRNA decay. Pathogenic variants in adjacent amino acid residues in exon 4 of *ACTA2* gene (N115 T, R116Q, N117S, N117I, R118Q) have been previously reported in families with TAAD which lie within the hydrophobic cleft of a-actin which acts as the binding determinant of several regulatory proteins and is thought to have a key role in actin polymerization [33–36]. These variants including Met121 occurred in evolutionarily, highly conserved amino acid residues (data not shown) which are more likely to contain pathogenic variants (UniProt sequence alignment tool against the genome of six species human/chimpanzee/mouse/rabbit/chicken/fish). The R118Q variant lies within the hydrophobic cleft of a-actin which acts as the binding site of several regulatory proteins [33]. The presence of p.Met121Ilefs*5 variant was also identified in both mother and sister of the affected son. The identification of the p.Met121Ilefs*5 variant in the apparently normal, non-symptomatic daughter, led to the recommendation of a follow-up, where a mild dilation of the ascending aorta with 4-5 mm increase in diameter was observed.

The non-synonymous substitution c.3234C > G might lead to changes in the protein function, concluding that this variant is likely responsible for causing the TAA phenotype. The p.Ile1078Met variant in MYH11 was also identified in evolutionarily, highly conserved amino acid residues which is more likely to contain pathogenic variants. The affected son was heterozygous for the p.Ile1078Met (c.3234C > G) variant which was also observed in the affected father and sister. The p.Ile1071Met variant was inherited from the father and the p.Met121Ilefs*5 variant was inherited from the mother.

Phenotype considerably varies among carriers of mutations in genes associated to TAA and familial occurrence of aortic dissections should always be investigated [37]. Therefore, there is a need for further genetic studies in cases of non-syndromic familial aneurysms so that patients with previous negative or inconclusive results on the diagnostic echo test may benefit from re-testing while avoiding unnecessary concerns in the non-carriers. This proof of principle theory has been demonstrated in our case report. The genetic investigation began with the affected son referred to our Institute. To the best of our knowledge, this is the first report of a familial TAAD case with presenting two novel variants, p.Met121Ilefs*5 and p.Ile1078Met in *ACTA2* and *MYH11* genes respectively. However, for the verification of these variants and their contribution in the development of TAA, further testing on additional samples from affected individuals is necessary. By looking at the family pedigree, from the father’s side, relatives have been contacted by the clinician and informed of the study undertaken and asked to contact the AMC at their convenience to have an assessment as well as to provide a blood sample. These will be used to screen for the p.Ile1078Met variant in *MYH11* gene in order to indicate the presence of an association with TAA. So far, the family has not communicated with the AMC. The heterozygous p.Ile1078Met variant in *MYH11* gene is a potentially disease-causing variant thus, further studies are needed to elucidate its role in pathogenicity. A limitation of this study is the absence of functional assay of the two identified variants to provide their functional relevance in TAA. Another limitation is that the study focused on the most prevalent genes that are related to TAA since these were included in the selected sequencing gene panel. Future investigations on whole exome sequencing could determine the spectrum of genetic variation and mutations associated with non-syndromic familial TAA in Cypriot patients.

In conclusion, we describe a Cypriot familial case with TAA. The screening process for the identification of further mutations and variants, in addition to the two novel variants in the *ACTA2* and *MYH11* genes identified in this report is ongoing. These results will provide a powerful tool for the early identification of non-syndromic familial TAAD patients in order to prevent devastating events by increasing the early screening and prophylactic treatments of affected family members.

## Additional file

Additional file 1: Genes associated with the development of TAAD. (PDF 264 kb)

## Abbreviations

ACMG: American College of Medical Genetics and Genomics
ACTA2: Smooth muscle cytoskeletal protein actin alpha(α)-2
AMC: American Medical Center
ANNOVAR: Annotate Variation
BWA: Burrows Wheeler Aligner
CT: Computed tomography
GATK: Genome Analysis Toolkit
GnomAD: Genome Aggregation Database
InDels: Insertions and Deletions
I-TASSER: Iterative Threading ASSEmbly Refinement
MPS: Massively Parallel Sequencing
MYH11: Myosin heavy chain 11
PDB: Protein Data Bank
PolyPhen2: Polymorphism Phenotyping
PROVEAN: Protein Variation Effect Analyzer
SAM: Sequence Alignment/Map
SIFT: Sorting Intolerant from Tolerant
SMC: Smooth muscle cells
SNV: Small Nucleotide Variants
TAA: Thoracic aortic aneurysm
TAAD: Thoracic aortic aneurysm and dissection
VEP: Variant Effect Predictor
VUS: Variant of Uncertain Significance
