# Supplementary material for: Novel variants in the ACTA2 and MYH11 genes in a Cypriot family with thoracic aortic aneurysms: a case report
Source: BMC Med Genet. 2018 Dec 7;19:208. doi: 10.1186/s12881-018-0728-0 (PMC6286578; doi:10.1186/s12881-018-0728-0)
Supplement: Supplementary file 1 — Genes associated with the development of TAAD. (PDF 264 kb) [file 12881_2018_728_MOESM1_ESM.pdf]

Genes associated with the development of TAAD

Gene	Protein
ACTA2	Smooth muscle α-actin
BGN	Biglycan
COL1A2	Collagen 1 α2 chain
COL3A1	Collagen 3 α1 chain
COL5A1	Collagen 5 α1 chain
COL5A2	Collagen 5 α2 chain
EFEMP2	Fibulin-4
ELN	Elastin
EMILIN1	Elastin microfibril interface 1
FBN1	Fibrillin-1
FBN2	Fibrillin-2
FLNA	Filamin A
FOXE3	Forkhead box 3
LOX	Lysyl oxidase
MAT2A	Methionine adenosyl-transferase II α
MFAP5	Microfibril-associated glycoprotein 2
MYH11	Smooth muscle myosin heavy chain
MYLK	Myosin light chain kinase
NOTCH1	NOTCH1
PRKG1	Type 1 cGMP-dependent protein kinase
SKI	Sloan Kettering proto-oncoprotein
SLC2A10	Glucose transporter 10
SMAD2	SMAD2
SMAD3	SMAD3
SMAD4	SMAD4
SMAD6	SMAD6
TGFB2	TGF-β2
TGFB3	TGF-β3
TGFBR1	TGF-β receptor type 1
TGFBR2	TGF-β receptor type 2
